# Characterization of *Miscanthus* cell wall polymers

**DOI:** 10.1111/gcbb.12538

**Published:** 2018-08-13

**Authors:** Judith Schäfer, Melinda Sattler, Yasir Iqbal, Iris Lewandowski, Mirko Bunzel

**Affiliations:** ^1^ Department of Food Chemistry and Phytochemistry, Institute of Applied Biosciences Karlsruhe Institute of Technology (KIT) Karlsruhe Germany; ^2^ Biobased Products and Energy Crops (340b), Institute of Crop Science University of Hohenheim Stuttgart Germany

**Keywords:** biomass, cell wall polysaccharides, cross‐links, lignin, Miscanthus

## Abstract

Efficient utilization of lignocellulosic *Miscanthus* biomass for the production of biochemicals, such as ethanol, is challenging due to its recalcitrance, which is influenced by the individual plant cell wall polymers and their interactions. Lignocellulosic biomass composition differs depending on several factors, such as plant age, harvest date, organ type, and genotype. Here, four selected *Miscanthus* genotypes (*Miscanthus sinensis*,* Miscanthus sacchariflorus*,* Miscanthus* × *giganteus*,* Miscanthus sinensis* × *Miscanthus sacchariflorus* hybrid) were grown and harvested, separated into stems and leaves, and characterized for their non‐starch polysaccharide composition and structures, lignin contents and structures, and hydroxycinnamate profiles (monomers and ferulic acid dehydrodimers). Polysaccharides of all genotypes are mainly composed of cellulose and low‐substituted arabinoxylans. Ratios of hemicelluloses to cellulose were comparable, with the exception of *Miscanthus sinensis* that showed a higher hemicellulose/cellulose ratio. Lignin contents of *Miscanthus* stems were higher than those of *Miscanthus* leaves. Considering the same organs, the four genotypes did not differ in their Klason lignin contents, but *Miscanthus* × *giganteus* showed the highest acetylbromide soluble lignin content. Lignin polymers isolated from stems varied in their S/G ratios and linkage type distributions across genotypes. *p*‐Coumaric acid was the most abundant ester‐bound hydroxycinnamte monomer in all samples. Ferulic acid dehydrodimers were analyzed as cell wall cross‐links, with 8‐5‐coupled diferulic acid being the main dimer, followed by 8‐O‐4‐, and 5‐5‐diferulic acid. Contents of *p*‐coumaric acid, ferulic acid, and ferulic acid dimers varied depending on genotype and organ type. The largest amount of cell wall cross‐links was analyzed for *Miscanthus sinensis*.

## 1 INTRODUCTION

There is an increasing interest in using biomass from fast growing non‐food perennial grasses as renewable resource. *Miscanthus*, a perennial C4 grass, is considered a leading candidate for bioenergy production due to its high biomass yield capacity, tolerance for temperate climates, and its ability to grow on marginal land. Currently, *Miscanthus* × *giganteus* is the only genotype that is commercially available in Europe. Introducing new genotypes are required in order to reduce biomass production costs, improve stress tolerance, and adapt biomass characteristics to user requirements (Lewandowski et al., 2016; Lewandowski, Clifton‐Brown, Scurlock, & Huisman, 2000).

Lignocellulosic biomass is made up of plant cell wall polymers. Their composition is complex and often determines biomass recalcitrance. Cell walls of grasses are mainly composed of cellulose, hemicelluloses (arabinoxylans and mixed‐linked β‐glucans in particular grasses), and lignin (Vogel, 2008). In addition, hydroxycinnamates, such as ferulic and *p*‐coumaric acids are bound to polysaccharides and lignin. Ferulic acid is usually attached to arabinoxylans and is responsible for the formation of cross‐links between polysaccharides, and polysaccharides and lignin, whereas *p*‐coumaric acid is usually bound to lignin (Bunzel, 2010; Ralph, 2010) with only minor amounts of *p*‐coumaric acid being attached to arabinoxylans (Allerdings, Ralph, Steinhart, & Bunzel, 2006). In order to obtain biochemicals and biofuels from lignocellulosic biomass, efficient pretreatment procedures, followed by enzymatic hydrolysis, and microbial fermentation processes are required (Rastogi & Shrivastava, 2017). Biomass pretreatment removes or alters lignin, a key factor of biomass recalcitrance, and loosens the cell wall polymer network. Enzymatic hydrolysis converts cellulose and hemicelluloses into monomers, which are fermented using various microorganisms. The efficiency of these processes varies considerably depending on biomass composition. Thus, cell wall composition data of various *Miscanthus* genotypes is crucial to select suitable genotypes for potential biochemical and biofuel production.

The gross composition of *Miscanthus* biomass, i.e., cellulose, hemicellulose, and lignin contents, has been described earlier (Arnoult & Brancourt‐Hulmel, 2015; da Costa et al., 2017; Hodgson et al., 2011; Lygin et al., 2011; Zhao et al., 2014). A few authors also report some structural details such as lignin monomer composition and/or lignin linkage type profiles (Cheng, Sorek, Zimmermann, Wemmer, & Pauly, 2013; Le Ngoc Huyen, Rémond, Dheilly, & Chabbert, 2010; Villaverde, Li, Ek, Ligero, & Vega, 2009), and acetylation degree of polysaccharides (Van der Weijde et al., 2017). However, these data are mostly limited to *Miscanthus* × *giganteus*. Studies on structural details of cell wall polymers such as lignin linkage type profiles and ferulic acid dehydrodimer contents specific to other *Miscanthus* genotypes are rarely available. In addition, published studies often describe the relationship between individual constituents and fermentation yields without analyzing the whole cell wall composition in detail. In general, polysaccharide contents mainly determine the fermentation yield. However, efficient biomass utilization is determined by the whole cell wall composition because structural features of cell wall polymers and polymer interactions also affect biomass recalcitrance. Lignin is a key factor in determining biomass recalcitrance, and the biomass needs to be pretreated to remove lignin. Besides lignin contents, the structural composition of lignin polymers may influence pretreatment processes, e.g. high portions of β‐O‐4‐ethers can be easily cleaved during pretreatment processes. Therefore, it is important to characterize all cell wall polymers of different *Miscanthus* genotypes in detail instead of limiting the analytical work to specific polymers to better evaluate the genotypes for biomass utilization. For this reason, we analyzed four *Miscanthus* genotypes for their cell wall composition, and all relevant polymers were characterized in detail.

## 2 MATERIALS AND METHODS

### 2.1 Chemicals

Heat‐stable α‐amylase Termamyl 120 L (from *Bacillus licheniformis*, 120 KNU/g), the protease Alcalase 2.5 L (from *Bacillus licheniformis*, 2.5 AU/g), and the amyloglucosidase AMG 300 L (from *Aspergillus niger*, 300 AGU/g) were kindly donated by Novozymes (Bagsvaerd, Denmark); the complex carbohydrase mixture Driselase (from Basidiomycetes sp.) was from Sigma‐Aldrich (St. Louis, MO). Chemicals used, including deuterated NMR solvents, were either from Sigma‐Aldrich, Roth (Karlsruhe, Germany), VWR (Radnor, PA, USA), or Alfa Aesar (Ward Hill, MA, USA).

### 2.2 Field trial description

*Miscanthus* material was harvested from a field trial established as a part of the European project OPTIMISC to compare 15 *Miscanthus* genotypes at six sites across Europe. A detailed description of the field trials and genetic material is provided in Kalinina et al. (2017). For this study four *Miscanthus* genotypes were selected from the location Stuttgart (lat. 48°74′N; long. 8°93′E). The field trial was planted from in‐vitro propagated plants on 22nd May 2012 on former arable land. Before planting out the plugs, the ground was prepared as follows: Weeds were removed with glyphosate, inversion ploughed and harrowed to produce a fine tilth. After planting, the plugs were watered to provide a good hydraulic contact between the soil and plug. The genotypes were established in randomized complete block design with three replications and each plot comprised of 5 × 5 m with 49 plants (plugs) per plot (resulting in a density close to 2 plants per m^2^).

Post‐planting herbicide was not applied in the first year, and weeds were controlled mechanically. In the first year (2012), fertilizer was applied at all the sites at rates of 44 and 110 kg/ha phosphorus (P) and potassium (K), respectively. No nitrogen (N) fertilizer was applied that year to avoid stimulating weed growth. From the second year onwards fertilizer was applied at a rate of 100 kg/ha P, 140 kg/ha K and 60 kg/ha N to ensure non‐limiting crop nutrition.

### 2.3 Genotype description

The selected genotypes belong to the species of *Miscanthus sinensis* (OPM‐11), *Miscanthus sacchariflorus* (OPM‐3), or their hybrids *Miscanthus* × *giganteus* (OPM‐9) and *Miscanthus sinensis* × *Miscanthus sacchariflorus* (OPM‐6; Table 1). The sterile clone *Miscanthus* × *giganteus* is a naturally occurring interspecific hybrid (*Miscanthus sinensis* × *Miscanthus sacchariflorus*) that has been used commercially in Europe for biomass production for over a decade and is therefore selected as standard reference clone. In general, *Miscanthus sinensis* interspecific hybrids have thinner and shorter stems than *Miscanthus sacchariflorus* and their hybrids, which combined lead to lower yields in trials with the scientific standard planting density of 20,000 plants/ha (Iqbal & Lewandowski, 2014). Additionally, lower yields of *Miscanthus sinensis* hybrids are caused by earlier senescence than *Miscanthus sacchariflorus*, which also impacts biomass composition (Iqbal et al., 2017). Genotypes OPM‐3, ‐6 and ‐11 stem from the *Miscanthus* breeding program led by the University of Aberystwyth. Over the past decade, it focused on producing interspecific *Miscanthus sinensis* × *M. sacchariflorus* hybrids with high yield, cold or other stress tolerance, and seed production (Clifton‐Brown et al., 2017). The standard genotype *Miscanthus* × *giganteus* was also provided by University of Aberystwyth.

**Table 1:** Selected genotypes for cell wall characterization. The samples were provided by University of Aberythwyth, UK

Genotype ID	Species	Chromosome number
OPM‐3	*Miscanthus sacchariflorus*	76
OPM‐6	*Miscanthus sinensis* × *Miscanthus sacchariflorus* hybrid	38
OPM‐9	*Miscanthus* × *giganteus*	57
OPM‐11	*Miscanthus sinensis* “Goliath”	57

### 2.4 Sample collection

For this study, biomass grown in the fifth year of the plantation was harvested on 20th March 2017, which is the preferred harvest time for *Miscanthus* biomass. Samples were harvested at the same time to minimize environmental effects on the cell wall composition. For biomass yield assessment samples were collected using manual cutters through harvesting the middle 2 m^2^ of a plot at a cutting height of ~5 cm. Sub‐samples were taken randomly from the 2 m^2^ cut and separated into leaves and stems. The flowers were considered as part of leaves. The collected samples were chopped and dried at 60°C for 48 hr in a cabinet dryer. The dried leave and stem fractions were milled through a cutting mill (SM 200‐Retsch, Haan, Germany) equipped with 1 mm sieve size.

### 2.5 Preparation of cell wall material

Preparative amounts of cell wall material were obtained using the principles of AOAC method 985.29 (Prosky et al., 1985) with minor modifications. Dried material was suspended with 1.5 ml of α‐amylase in 200 ml of sodium phosphate buffer (0.08 M, pH 6.2) and stirred for 20 min at 92°C. Samples were cooled to room temperature, the pH was adjusted to 7.5 with 0.275 M NaOH, protease (600 µl) was added, and the samples were incubated for 30 min at 60°C. After cooling the samples to room temperature, the pH was adjusted to 4.5 using 0.325 M HCl. Amyloglucosidase (700 µl) was added, and the samples were incubated for 30 min at 60°C. A fourfold amount (v/v) of ethanol (99%) was added, and precipitation was completed overnight. Following centrifugation, residues were washed three times with 78% ethanol and acetone, and dried at 60°C in a vacuum oven. The isolated cell wall material was further Soxhlet extracted for 6 hr each with ethanol, ethyl acetate, and *n*‐hexane.

### 2.6 Polysaccharide analysis

Monomer composition of cell wall polysaccharides was analyzed after H_2_SO_4_ hydrolysis (Saeman, Bubl, & Harris, 1945); Klason lignin filtrates (see Lignin analysis) were used for this purpose. The filtrates were diluted and analyzed by high‐performance anion exchange chromatography with pulsed amperometric detection (HPAEC‐PAD; Wefers, Gmeiner, Tyl, & Bunzel, 2015). Methylation analysis (Pettolino, Walsh, Fincher, & Bacic, 2012) was used to determine interunit linkages of the polysaccharide constituents as detailed in Wefers et al. (2015). In brief, methylation was performed in DMSO/NaOH using 1 ml of methyl iodide followed by dichloromethane extraction. The methylation step was performed twice. Hydrolysis of the methylated polysaccharides was performed using 2 ml of 2 M trifluoroacetic acid for 90 min at 120°C, followed by reduction with 20 mg of NaBD_4_, acetylation with acetic anhydride (3 ml) and 1‐methylimidazole (450 µl), and extraction of the partially methylated alditol acetates (PMAAs) into dichloromethane. PMAAs were identified by GC‐MS and semiquantitatively determined by GC‐FID using molar response factors (Sweet, Shapiro, & Albersheim, 1975). The GC‐MS system (GC‐MS QP2010 SE, Shimadzu, Kyoto, Japan) was equipped with a DB‐225MS column (30 m × 0.25 mm i.d., 0.25 µm, Agilent Technologies, Santa Clara, CA, USA). The following conditions were used: Initial column temperature 140°C, held for 1 min, ramped at 20°C/min to 170°C, ramped at 2°C/min to 200°C, ramped at 20°C/min to 220°C, and held for 20 min. Helium was used as carrier gas at 40 cm/s. The injection temperature was 220°C. GC‐FID analysis was performed on a DB‐225 column (30 m × 0.25 mm i.d., 0.25 µm film thickness, Agilent Technologies) with the same conditions as described above. To determine the ratio of PMAAs resulting from 1,2,4‐ and 1,3,4‐linked xylopyranose units a Rtx‐5MS (30 m × 0.25 mm × 0.25 µm, Restek, Bad Homburg, Deutschland; GC‐MS) and a DB‐5 (30 m × 0.25 mm × 0.25 µm, Agilent Technologies; GC‐FID) column were used applying the following conditions: initial temperature 140°C, held for 2 min, ramped at 1°C/min to 180°C, held for 5 min, ramped at 10°C/min at 300°C, and held for 5 min. Helium was used as carrier gas at 40 cm/s, and the injection temperature was 250°C.

### 2.7 Determination of lignin contents

Lignin contents were determined as Klason lignin (Adams, 1965; Theander & Westerlund, 1986) and as acetyl bromide soluble lignin (ABSL; Hatfield, Grabber, Ralph, & Brei, 1999; Iiyama & Wallis, 1990; Johnson, Moore, & Zank, 1961) as detailed in Bunzel, Schüßler, and Tchetseubu Saha (2011) and Bunzel, Seiler, and Steinhart (2005). To determine Klason lignin contents, cell wall material was suspended in 5 ml of 12 M H_2_SO_4_, and the suspensions were stirred on ice for 30 min and additionally for 2 hr at room temperature. The acid was diluted with 32.5 ml of H_2_O, and hydrolysis was performed at 100°C for 3 hr. After filtration through glass microfiber filters (1.6 µm; Whatman, Little Chalfont, England), the residues were washed acid free and dried at 60°C. The filtrates were used for monosaccharide determination (see Polysaccharide analysis). Klason lignin contents were corrected for residual ash and protein contents. Ash contents were analyzed gravimetrically after incineration of the samples at 550°C for 5 hr. Nitrogen contents of the Klason lignin samples were determined after Kjeldahl digestion. Liberated ammonium‐N was analyzed using an ammonium selective electrode (Thermo Scientific, Waltham, MA). Protein contents were calculated using the general conversion factor 6.25.

In preparation of the ABSL assay, cell wall material was ball‐milled using a PM100 planetary ball mill with zirconium dioxide balls (Retsch, Haan, Germany; 1 g, 600 rpm, 3.25 hr, 5 min interval with 5 min break between millings), following enzyme digestion using the carbohydrate mixture Driselase (60 mg of Driselase and 100 ml of H_2_O per gram of milled sample material). Enzyme‐digested material (20–25 mg) was suspended in 4 ml of 25% acetyl bromide in glacial acetic acid and stirred for 2 hr at 50°C. The solution was diluted with 12 ml of glacial acetic acid. Sample solution (1 ml or 0.5 ml), 2.5 ml of glacial acetic acid, 1.5 ml of 0.3 M NaOH, and 0.5 ml of a 0.5 M hydroxylamine hydrochloride solution were mixed, the volume was adjusted to 10 ml with glacial acetic acid, and the absorbance of the mixture was read at 280 nm on a Jasco V‐550 spectrometer (Jasco, Groß‐Umstadt, Germany). Lignin contents were calculated using an absorption coefficient of 20 ml cm^−1^ mg^−1^ (Iiyama & Wallis, 1990).

### 2.8 Lignin structural analysis

Both monomer composition and linkage type distribution of lignin polymers were determined using 2D‐NMR analyses. To determine monolignol ratios, ball‐milled cell wall material (1 g, 600 rpm, 3.25 hr, 5 min interval with 5 min break between millings) was directly swollen in the NMR tube using DMSO‐*d_6_*/pyridine‐*d_5_* (4:1; Kim & Ralph, 2010). The samples were sonicated in an ultrasonic bath until the gel became homogeneous. HSQC spectra were acquired on a 500 MHz Bruker Avance NMR spectrometer (Rheinstetten, Germany) equipped with a Prodigy cryoprobe. A standard Bruker HSQC‐pulse program (adiabatic pulse) was used applying the following parameters (Mansfield, Kim, Lu, & Ralph, 2012): 1,024 datapoints in f_2_ (acquisition time = 85 ms) and 512 datapoints in f_1_ (acquisition time = 9 ms), relaxation delay (D1) 0.5 s. Processing was done using Gaussian apodization (LB: −0.1, GB: 0.001) in f_2_ and sine squared apodization (SSB: 2) in f_1_. Semiquantitative analysis of the monolignols was performed by volume integration of specific signals (Kim & Ralph, 2010; Mansfield et al., 2012), i.e. carbon‐2 correlations were used for guaiacyl (G) units (intensities were doubled), and carbon‐2/carbon‐6 correlations were used for syringyl (S) units.

Isolation of lignin polymers was necessary to determine linkage type distributions (Bunzel & Ralph, 2006; Schäfer, Wagner, Trierweiler, & Bunzel, 2016). Ball‐milled cell wall material (see above) was enzyme‐digested (60 mg of Driselase and 100 ml of H_2_O per gram of milled sample material), and heated with dioxane/0.01 M aqueous HCl (85/15, v/v) under nitrogen. The suspension was filtered, concentrated, and dissolved lignin was precipitated overnight in acidified water (pH 2). Following centrifugation, lignin was washed with water and lyophilized. Isolated lignin preparations were acetylated using pyridine and acetic anhydride (1:1, v/v). Acetylation reagents were evaporated, and acetylated lignins were dissolved in 500 µl of acetone‐*d_6_* and analyzed by 2D‐NMR spectroscopy (HSQC experiment, adiabatic pulse). The following parameter were used: 2,048 datapoints in *f*_2_ (acquisition time = 170 ms) and 512 datapoints in f_1_ (acquisition time = 9 ms), relaxation delay (D1) 1 s. Processing was done using Gaussian apodization (LB: −1, GB: 0.001) in f_2_ and sine squared apodization (SSB: 2) in *f*_1_. Linkage type determination was achieved by integration of contours representing α‐carbon‐proton correlations (Bunzel & Ralph, 2006; Ralph, Ralph, & Landucci, 2009), however, without using correction factors. The integral resulting from α‐carbon‐proton correlations of resinol units was halved.

### 2.9 Determination of phenolic monomers and ferulic acid dehydrodimers and ‐trimers

Cell wall material (15–75 mg) was saponified (5 ml of 2 M NaOH) for 18 hr under nitrogen and protected from light (Dobberstein & Bunzel, 2010). Following acidification, the internal standard compounds *trans*‐*o*‐coumaric acid (20–150 µg, depending on the sample) and 5–5(methylated)‐dehydrodiferulic acid (0.5–3 µg, depending on the sample) were added. Extraction was performed three times using 2 ml of diethylether. Extracts were dried under nitrogen, redissolved in 0.5 ml of tetrahydrofuran/H_2_O (50:50, v/v), and analyzed by HPLC‐DAD on a 250 mm × 4.6 mm, 5 µm Luna phenylhexyl column (Phenomenex, Torrance, CA; Dobberstein & Bunzel, 2010). Quantitation of phenolic monomers was performed at 308 nm for *trans*‐*p*‐coumaric‐acid, at 294 nm for *cis*‐*p*‐coumaric acid, at 321 nm for *trans*‐ferulic acid, at 310 nm for *cis*‐ferulic acid, and at 323 nm for *trans*‐*o*‐coumaric acid. Quantitation of ferulic acid dehydrodimers and ‐trimers was conducted at 280 nm using the correction factors described in literature (Dobberstein & Bunzel, 2010). Because the concentration range of some diferulic acids (DFA) exceeded the concentration range tested by Dobberstein and Bunzel (2010), the linear response was tested and confirmed for the concentrations measured in the samples.

## 3 RESULTS AND DISCUSSION

To study cell wall structural details, four *Miscanthus* genotypes were selected. The main criterion for the selection of the four genotypes tested in this study was a high dry matter yield potential and contrasting biomass composition (Kalinina et al., 2017).

### 3.1 Polysaccharide composition

Being a monocotyledonous plant, *Miscanthus* cell wall polysaccharide composition was described to show typical traits of grasses in the past (De Souza et al., 2015; Domon et al., 2013; Le Ngoc Huyen et al., 2010; Lygin et al., 2011). Generally, type II primary cell walls of grasses are mainly composed of cellulose and arabinoxylans as predominant hemicellulosic polymers. Other hemicelluloses besides arabinoxylans as well as pectins are present in small amounts only (Carpita & Gibeaut, 1993; Vogel, 2008). However, detailed structural analyses of *Miscanthus* cell wall polysaccharides are rarely reported in literature (da Costa et al., 2017).

Here, cell wall polysaccharides of leaves (including flowers) and stems from different *Miscanthus* genotypes were characterized by monosaccharide analysis after H_2_SO_4_ hydrolysis and by methylation analysis (Tables 2 and 3). The dominant neutral sugars were glucose (52.6 mol%–63.0 mol%), xylose (34.1 mol%–40.4 mol%), and arabinose (2.6 mol%–6.6 mol%), suggesting cellulose and arabinoxylans as main cell wall polysaccharides. Methylation analysis data showed that most of the glucopyranose units were 1,4‐linked, confirming cellulose as dominant polysaccharide. H_2_SO_4_ hydrolysis of stem polysaccharides resulted in slightly higher portions of glucose compared to glucose portions of leaf polysaccharides, suggesting higher cellulose portions in *Miscanthus* stems, which was already shown for different *Miscanthus sinensis* genotypes (Van der Weijde et al., 2017). In addition, low portions of PMAAs representing 1,3‐linked glucopyranose units were identified in both stems and leaves, suggesting either mixed‐linked β‐glucans and/or callose in *Miscanthus* cell walls. Also, the identification of low portions of PMAAs representing 1,4,6‐linked glucopyranose units demonstrates low amounts of xyloglucans in all samples analyzed.

**Table 2:** Polysaccharide monomer composition (mol%) of four *Miscanthus* genotypes (Table 1) separated into stems and leaves analyzed after H_2_SO_4_ hydrolysis

mol% (g/100 g dry weight)	Leaves				Stems			
	OPM‐3	OPM‐6	OPM‐9	OPM‐11	OPM‐3	OPM‐6	OPM‐9	OPM‐11
Arabinose	5.9 ± 0.1 (3.4 ± 0.2)	6.5 ± 0.1 (3.6 ± 0.1)	6.5 ± 0.1 (5.0 ± 0.2)	6.6 ± 0.01 (5.6 ± 0.1)	2.6 ± 0.2 (1.6 ± 0.2)	3.5 ± 0.1 (2.1 ± 0.02)	2.9 ± 0.1 (2.2 ± 0.2)	4.5 ± 0.01 (3.2 ± 0.1)
Glucose	56.0 ± 0.1 (38.8 ± 2.1)	57.8 ± 0.1 (38.1 ± 0.8)	56.3 ± 0.5 (52.3 ± 2.2)	52.6 ± 0.4 (53.3 ± 0.9)	62.3 ± 0.4 (44.5 ± 2.4)	60.6 ± 0.4 (44.4 ± 0.6)	63.0 ± 0.6 (56.5 ± 7.1)	55.1 ± 0.5 (47.3 ± 0.5)
Xylose	38.1 ± 0.1 (22.1 ± 1.2)	35.7 ± 0.1 (19.7 ± 1.3)	35.9 ± 0.5 (27.8 ± 1.4)	39.7 ± 0.5 (33.6 ± 0.3)	35.1 ± 0.6 (20.8 ± 0.6)	35.9 ± 0.4 (22.0 ± 0.5)	34.1 ± 0.5 (25.5 ± 3.3)	40.4 ± 0.5 (29.0 ± 0.9)
Galactose			1.4 ± 0.03 (1.3 ± 0.1)	1.2 ± 0.03 (1.2 ± 0.04)				

Notes

Galactose was detected in leaves of OPM‐3 and OPM‐6, and in the stems of all genotypes but concentrations were below the calibration range. Monosaccharide yields (g/100 g dry weight) of *Miscanthus* stems and leaves liberated after H_2_SO_4_ hydrolysis are given in parentheses. The calculated monosaccharide yields cannot easily be referred to the total carbohydrate contents due to varying susceptibility of glycosidic linkages to acid hydrolysis, and due to varying stability of the liberated monosaccharides in an acidic environment. Therefore, the calculated amounts simply represent monosaccharide yields liberated from polysaccharides after H_2_SO_4_ hydrolysis. *n* = 3.

**Table 3:** Ratios (mol%) of partially methylated alditol acetates (PMAAs) resulting from methylation analysis of *Miscanthus* leave and stem cell wall material (*n* = 1)

PMAA (mol%)	Leaves				Stems			
	OPM‐3	OPM‐6	OPM‐9	OPM‐11	OPM‐3	OPM‐6	OPM‐9	OPM‐11
t‐Glc*p*	1.7	2.1	1.7	1.5	2.0	1.5	1.2	1.1
1,3‐Glc*p*	2.0	1.9	2.2	1.9	1.6	1.3	1.1	1.2
1,4‐Glc*p*	45.2	45.7	47.2	41.1	46.6	42.3	44.3	39.3
1,4,6‐Glc*p*	0.9	1.0	0.9	1.0	0.8	0.8	0.9	0.8
t‐Xyl*p*	2.1	2.2	1.9	1.7	1.4	1.5	1.1	1.4
1,4‐Xyl*p*	28.3	22.3	22.1	24.1	32.2	26.9	26.5	27.6
1,2,4‐Xyl*p* a	1.0	1.2	1.2	1.2	0.7	1.1	1.1	1.2
1,3,4‐Xyl*p* a	6.8	7.1	7.3	7.3	4.0	5.0	4.4	5.8
1,2,3,4‐Xyl*p* b	2.8	5.7	4.6	11.0	5.1	13.5	14.6	14.9
t‐Ara*f*	6.3	7.4	7.8	6.5	4.8	5.5	4.2	6.0
1,2‐Ara*f*	0.7	0.8	0.6	0.5	d	d	d	d
1,3‐Ara*f*	d	0.5	0.5	0.6	d	d	d	d
1,5‐Ara*f*	1.4	1.5	1.2	1.0	0.8	0.6	0.6	0.7
t‐Gal*p*	1.0	0.7	0.9	0.6	d	d	d	d

Notes

t: terminal; *p*: pyranose; *f*: furanose; Glc: glucose; Xyl: xylose; Gal: galactose; Ara: arabinose; d: detected.

a Ratios of 1,2,4‐ and 1,3,4‐xylopyranoses were determined on a DB‐5 column due to coelution of this two PMAAs on the DB‐225 column.

b Overestimation possible due to undermethylation.

As expected, arabinoxylans were found to be the second most abundant polysaccharides. Arabinoxylans consist of a backbone of 1,4‐linked xylopyranose units that can be substituted with arabinofuranose units in position *O*‐2 and/or *O*‐3. The majority of acid liberated xylose appears to be 1,4‐linked. Substitution of the 1,4‐linked xylose backbone in position *O*‐2 and/or *O*‐3 was confirmed by identification of PMAAs resulting from 1,2,4‐, 1,3,4‐, and 1,2,3,4‐linked xylopyranose units. However, PMAAs representing 1,2,3,4‐linked xylopyranose units may also, at least partially, result from undermethylation, thereby potentially overestimating the portions of fully branched xylose units. Higher arabinose/xylose ratios of leaf polysaccharides (0.15–0.19) compared to stem polysaccharides (0.07–0.11) suggest a higher xylan backbone substitution of arabinoxylans from *Miscanthus* leaves. Several authors report arabinose/xylose ratios ranging from about 0.1 to 0.3 for *Miscanthus* plants, depending on the genotype (Domon et al., 2013; Lygin et al., 2011). Le Ngoc Huyen et al. (2010) analyzed lower arabinose/xylose ratios in *Miscanthus* internodes (ratios of about 0.07) compared to green leaves (ratios of about 0.22). Substitution of the xylose backbone in position *O*‐3 seems to be preferred in both stems and leaves, because PMAAs representing 1,3,4‐xylopryanose units were found in higher portions than PMAAs representing 1,2,4‐xylopyranose units (1,3,4‐/1,2,4‐xylopyranose ratios of 5.9–6.8 for leaves, and 4.0–5.7 for stems). Besides these major linkages, low portions of several PMAAs were identified that may result from complex arabinoxylan side chains. In general, the xylan backbone is substituted with single arabinofuranose units; however, oligomeric side chains are reported in literature, e.g. arabinofuranose units can be additionally substituted in position *O*‐2 with xylopyranose and/or more complex galactose containing di‐and trisaccharides (Allerdings et al., 2006; Saulnier, Vigouroux, & Thibault, 1995). In addition, PMAAs representing 1,2‐linked arabinofuranose units may result from oligomeric arabinofuranose side chains (Mazumder & York, 2010; Verbruggen et al., 1998). PMAAs representing 1,2‐arabinofuranose units were analyzed for both *Miscanthus* stems and leaves with slightly higher portions in the leaves. The identified small portions of *t*‐galactopyranose units may also result from complex arabinoxylan side chains such as α‐L‐galactopyranosyl‐(1→2)‐β‐D‐xylopyranosyl‐(1→2)‐5‐*O*‐*trans*‐feruloyl‐L‐arabinofuranose (Allerdings et al., 2006). Terminal xylopyranose units may either result from arabinoxylan side chains, i.e. β‐D‐xylopyranosyl‐(1→2)‐5‐*O*‐*trans*‐feruloyl‐L‐arabinofuranose (Allerdings et al., 2006), or from the non‐reducing end of the xylan backbone. PMAAs suggesting arabinose units that are substituted in position *O*‐3 were also identified in low portions. These structural units were reported to exist as arabinoxylan side chains in psyllium husk (Fischer et al., 2004). The identification of 1,5‐arabinofuranose units by methylation analysis in cereal grains is often traced back to arabinoxylan side chains. However, 1,5‐linked arabinofuranose units are more likely to arise from traces of pectic arabinans. To the best of our knowledge, no convincing data exist demonstrating 1,5‐linked arabinofuranose containing arabinoxylan side chains.

Polysaccharide characterization analyses reveal differences among the *Miscanthus* genotypes studied. Both leaf and stem polysaccharides of genotype OPM‐11 had higher portions of xylose (39.7 mol% for leaves and 40.4 mol% for stems) and less portions of glucose (52.6 mol% for leaves and 55.1 mol% for stems) than the other genotypes, suggesting higher portions of arabinoxylans but lower portions of cellulose for OPM‐11 cell wall polysaccharides compared to those of genotypes OPM‐3, OPM‐6, and OPM‐9. Hodgson et al. (2011) showed that *Miscanthus* × *giganteus* and *Miscanthus sacchariflorus* genotypes contain higher contents of cellulose and lower contents of hemicelluloses than the *Miscanthus sinensis* genotypes, too. The xylan substitution degree was comparably low among the *Miscanthus* genotypes analyzed here as demonstrated by similar, low arabinose/xylose ratios.

It is important to note, that polysaccharide characterization data do not represent absolute amounts of polysaccharides in the lignocellulosic biomass because the principles of the methods used in this study do not allow for a quantitative polysaccharide analysis. This is mostly due to varying susceptibility of glycosidic linkages to acid hydrolysis, and due to varying stability of the liberated monosaccharides in hot acidic solutions. However, calculated monosaccharide amounts liberated on a dry weight basis after H_2_SO_4_ hydrolysis can be found in Table 2.

Polysaccharide contents determine theoretical yields of fermentable monosaccharides. Efficient biomass utilization requires extensive hydrolysis of both cellulose and hemicelluloses. However, the efficiency of polysaccharide hydrolysis is affected, among others, by the overall biomass composition and interactions of cell wall polymers. The degradability of cellulose to glucose units is influenced by different factors, including its crystallinity and its interactions within the cell wall network (Chang & Holtzapple, 2000; Puri, 1984). Hemicelluloses are often described to act as physical barriers that limit enzymatic hydrolysis of cellulose (Zhao, Zhang, & Liu, 2012). In general, different pretreatments are used to solubilize hemicelluloses (and to reduce lignin contents), which enhance enzymatic degradability of cellulose (Yang, Chen, Chen, Wang, & Sun, 2015). However, hemicelluloses were also found to be positively associated to cell wall degradability and saccharification efficiency, depending on their structural features (Li et al., 2013; Van der Weijde et al., 2017; Xu et al., 2012). According to those studies, the higher hemicellulose/cellulose ratio of OPM‐11 may positively affect the polysaccharide degradability of this genotype. Hemicelluloses such as arabinoxylans are efficiently hydrolyzed using acidic pretreatments. However, liberated sugars are not completely stable and enzyme inhibitors such as furfural derivatives may be formed (Palmqvist & Hahn‐Hägerdal, 2000). In addition, both interactions of cell wall polymers and lignin contents may have greater impact on biomass utilization than simple hemicellulose to cellulose ratios.

### 3.2 Lignin contents and structures

**Figure 1:**
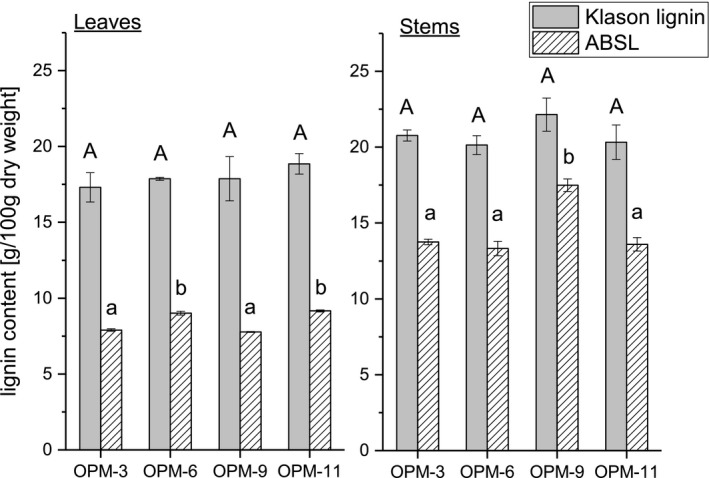
Klason lignin and acetylbromide soluble lignin (ABSL) contents of different *Miscanthus* genotypes (Table 1) separated into stems and leaves. Means labeled with different letters are statistically different (ANOVA, Tukey test, *α* = 0.05, leaves and stems are tested separately).* n* = 3

Lignin contents of *Miscanthus* leaves and stems were determined as both Klason lignin and ABSL. Both methods have limitations in analyzing absolute lignin contents, which also becomes obvious from the different lignin contents analyzed here using these methods (Figure 1). Klason lignin is measured gravimetrically as an acid insoluble residue and can be overestimated due to non‐hydrolyzed non‐lignin compounds such as proteins, waxes, and cutin (Bunzel et al., 2011). To minimize these effects, dried plant samples were Soxhlet extracted using different solvents, and the Klason lignin preparations were corrected for residual ash and protein contents as suggested by most protocols. ABSL is measured spectrophotometrically at 280 nm after solubilization, and may be overestimated due to polysaccharide‐bound phenolic compounds that absorb at 280 nm, too. For this reason, cell wall material was enzyme digested using a cell wall polysaccharide degrading enzyme mixture. However, ABSL contents may also be underestimated due to incomplete solubility of the lignin polymer in acetyl bromide and glacial acetic acid. Thus, comparison of absolute lignin contents with literature data is often difficult due to different methods and/or protocols used for lignin determination.

As expected, Klason lignin and ABSL contents of *Miscanthus* stems were higher than lignin contents of *Miscanthus* leaves. Klason lignin contents ranged from 20.14 ± 0.62 g/100 g dry weight to 22.14 ± 1.10 g/100 g dry weight in cell wall material from *Miscanthus* stems and from 17.31 ± 0.96 g/100 g dry weight to 18.85 ± 0.67 g/100 g dry weight in cell wall material from *Miscanthus* leaves. ABSL contents ranged from 13.32 ± 0.47 g/100 g dry weight to 17.49 ± 0.41 g/100 g dry weight in cell wall material from *Miscanthus* stems and from 7.78 ± 0.03 g/100 g dry weight to 9.16 ± 0.07 g/100 g dry weight in cell wall material from *Miscanthus* leaves. Klason lignin contents of the different genotypes were not statistically different within the two groups (leaves and stems; ANOVA, Tukey test, α = 0.05). In contrast, differences in the ABSL contents of the analyzed genotypes are statistically significant within the stem and leave fractions (ANOVA, Tukey test, *α* = 0.05). In the leave fraction, genotypes OPM‐6 and OPM‐11 had slightly higher ABSL contents than OPM‐3 and OPM‐9. In the stem fraction, which is the quantitatively dominant fraction, the highest ABSL content was analyzed for OPM‐9. Considering leave/stem ratios of about 0.20 (OPM‐3, OPM‐9), 0.31 (OPM‐11), and 0.48 (OPM‐6) it can be suggested that *Miscanthus* plants of OPM‐9 contain the highest ABSL contents among the genotypes studied here.

The recalcitrance of plant biomass to hydrolysis is commonly associated with increased lignification (Chen & Dixon, 2007). In general, lignin acts as a physical barrier and reduces the accessibility of polysaccharides to chemical and enzymatic hydrolysis (De Souza et al., 2015; Zhao et al., 2012). In addition, the efficiency of enzymatic saccharification is impeded because lignin irreversibly adsorbs hydrolytic enzymes (Berlin et al., 2006; Jørgensen, Kristensen, & Felby, 2007). In general, different pretreatment methods are used to remove lignin and enhance biomass digestibility (Mohapatra, Mishra, Behera, & Thatoi, 2017; Sun & Cheng, 2002). Therefore, low lignin contents are favorable to apply modest pretreatment conditions and efficient biomass saccharification. Thus, the high ABSL content of *Miscanthus* genotype OPM‐9 may contribute to higher recalcitrance of this genotype.

Besides lignin contents, structural composition of lignin polymers is suggested to play an important role for efficient biomass saccharification (Li, Pu, & Ragauskas, 2016; Simmons, Loqué, & Ralph, 2010). Lignin is a complex polymer, which is mainly composed of three monomers: *p*‐coumaryl, coniferyl, and sinapyl alcohol. These monomers form *p*‐hydroxyphenyl (H), G‐, and S‐units through oxidative radical coupling (Ralph et al., 2004). However, lignin structures vary among plant species, tissues, cell types, and maturation stage of the plants, and other phenolic compounds may be incorporated into the polymer, too.

Using whole cell wall 2D‐NMR analysis, lignins of *Miscanthus* leaves were characterized as being rich in G‐units (81.4%–92.1%) with lower portions of S‐units (7.9%–18.6%), resulting in S/G ratios of 0.09–0.23. In contrast, the portions of S‐units (25.0%–41.1%) were higher in *Miscanthus* stems (S/G ratios 0.33–0.70; Table 4). In literature, a few authors describe the monolignol composition using NMR spectroscopy to analyze whole cell wall material and milled wood lignins from *Miscanthus* × *giganteus* plants (Cheng et al., 2013; El Hage et al., 2009; Villaverde et al., 2009). Analyzed S/G ratios of about 0.7–0.8 are in agreement with our data. El Hage et al. (2009) and Cheng et al. (2013) also determined small portions of H‐units (about 5%). Here, small signals were detected in the particular region of the spectra where the carbon‐2/carbon‐6 correlations of H‐units usually appear. However, due to the difficult, not fully unambiguous assignment of the characteristic signal (Kim et al., 2017), H‐units were not determined.

**Table 4:** Monomer composition of lignin polymers from different genotypes of *Miscanthus* separated into leaves and stems (*n* = 3). Means, within the last column, labeled with different letters are statistically different (ANOVA, Tukey test, α = 0.05, leaves and stems are tested separately). G, guaiacyl; S, syringyl

	%S	%G	S/G
*Leaves*			
OPM‐3	8.5 ± 1.5	91.5 ± 1.5	0.09 ± 0.02^a^
OPM‐6	18.6 ± 1.8	81.4 ± 1.8	0.23 ± 0.03^b^
OPM‐9	7.9 ± 2.6	92.1 ± 2.6	0.09 ± 0.03^a^
OPM‐11	16.5 ± 1.8	83.5 ± 1.8	0.20 ± 0.03^b^
*Stems*			
OPM‐3	41.1 ± 2.0	58.9 ± 2.0	0.70 ± 0.06^a^
OPM‐6	33.5 ± 2.7	66.5 ± 2.7	0.51 ± 0.06^b^
OPM‐9	35.0 ± 0.7	65.0 ± 0.7	0.54 ± 0.02^b^
OPM‐11	25.0 ± 3.5	75.0 ± 3.5	0.33 ± 0.06^c^

Within the leave and stem fractions, different monolignol compositions were observed for the different genotypes. The S/G ratio of lignins from OPM‐3 stems (0.70) was significantly higher than the S/G ratios of lignins from the other genotypes (0.33–0.54; ANOVA, Tukey test, *α* = 0.05). The lowest S/G ratio was analyzed for lignins from OPM‐11 stems (0.33). The S/G ratios of lignins from OPM‐6 and OPM‐9 stems were similar (0.51 and 0.54). The monolignol composition of lignins from leaves of genotypes OPM‐6 and OPM‐11 (S/G ratios: 0.23 and 0.20) differs significantly from the monolignol composition of lignins from leaves of OPM‐3 and OPM‐9 (S/G ratio: 0.09). Considering the low leave/stem ratios, it can be suggested that lignins from *Miscanthus* plants of genotype OPM‐3 contain the highest portions of sinapyl alcohol.

Data about linkage types in *Miscanthus* lignins are rarely available in literature (Cheng et al., 2013; Villaverde et al., 2009). In general, β‐O‐4‐ethers are described as main linkage type, followed by β‐β‐, and β‐5‐linkages. In addition, Villaverde et al. (2009) found traces of β‐1‐linkages, whereas Cheng et al. (2013) determined comparably high portions of dibenzodioxocin structures (about 24%) by analyzing *Miscanthus* biomass using 2D‐NMR analysis. In the current study, linkage type profiles were determined by 2D‐NMR after isolation of lignins from the plant materials (Table 5). The most abundant linkage types in *Miscanthus* leaves were β‐aryl‐ether (β‐O‐4) (60.5%–65.7%), followed by phenylcoumaran units (β‐5) (11.3%–14.9%), β‐1‐linkages (sum of traditional β‐1 and spirodienone units) (9.8%–14.4%), dibenzodioxocin (4.9%–7.6%), and resinol units (0.3%–2.3%). β‐O‐4‐Linkages were the predominant linkage type in *Miscanthus* stems (59.7%–76.7%), too, followed by β‐1‐linkages (sum of traditional β‐1 and spirodienone units) (15.4%–18.7%), phenylcoumaran units (β‐5) (3.4%–10.7%), dibenzodioxocin (1.6%–4.9%), and resinol units (1.3%–1.7%). The “traditional β‐1 units” are suggested to arise from spirodienone units under the acidic conditions of the lignin isolation process (Ralph & Landucci, 2010; Zhang & Gellerstedt, 2001). Therefore, portions of both structural units can be summarized. The portion of β‐β‐coupled units may be underestimated. β‐β‐Coupling of monolignols does not only result in typical resinol units but also in tetrahydrofuran structures. Tetrahydrofuran structures are formed from acetylated monolignols or preformed *p*‐coumaric acid/*p*‐hydroxybenzoic acid monolignol conjugates (Lu & Ralph, 2008; Lu et al., 2015). As described below, *Miscanthus* cell walls contain high contents of *p*‐coumaric acid, which is suggested to be mostly attached to lignin (Ralph, 2010). Therefore, tetrahydrofuran units are likely; however, we were not able to identify characteristic signals in the NMR spectra.

**Table 5:** Linkage type profiles of isolated lignins from different *Miscanthus* genotypes separated into leaves and stems (*n* = 1)

	%A^a^	%B	%C	%D	%*SD*	%F^1^	%X1
*Leaves*							
OPM‐3	60.5	14.9	2.3	7.6	2.7	7.1	4.9
OPM‐6	65.7	11.3	0.8	5.5	3.8	10.6	2.4
OPM‐9	64.6	12.5	1.6	4.9	3.0	10.7	2.8
OPM‐11	61.1	12.2	0.3	6.6	4.5	9.9	5.5
*Stems*							
OPM‐3	66.9	8.1	1.4	3.3	3.7	14.4	2.2
OPM‐6	65.4	9.1	1.3	2.3	2.8	15.9	3.1
OPM‐9	76.7	3.4	2.0	1.6	3	12.4	0.9
OPM‐11	59.7	10.7	1.7	4.9	4.2	14.4	4.4

Note

Ratios were semiquantitatively calculated by volume integration of characteristic signals. A, β‐aryl‐ether (β‐O‐4); B, phenylcoumaran (β‐5); C, resinol (β‐β); D, dibenzodioxocin (5–5/β‐O‐4); *SD*, spirodienone (β‐1/α‐O‐α); F, traditional β‐1; X1, cinnamyl endgroups.

a Under‐/overestimation possible due to partial signal overlapping.

Only slight differences in linkage type distributions were observed for lignins isolated from *Miscanthus* leaves across genotypes. In contrast, lignins isolated from *Miscanthus* stems of OPM‐9 and OPM‐11 showed different linkage type profiles than those from stems of genotypes OPM‐3 and OPM‐6, which have similar linkage type distributions. Lignins isolated from stems of OPM‐11 had the lowest portion of β‐O‐4 linkages (59.7%). In contrast, the highest portion of β‐O‐4 linkages (76.7%) was observed for lignins isolated from OPM‐9 stems. In addition, phenylcoumaran units (3.4%) were less abundant in this genotype compared to phenylcoumaran portions of the other genotypes (8.1%–10.7%).

β‐O‐4‐Ethers are more labile than carbon‐carbon bonds (e.g., β‐5, β‐β, β‐1) and can be cleaved during pretreatment of the biomass, with the extent being dependent on the pretreatment type (Pu, Hu, Huang, & Ragauskas, 2015). For this reason, genotypes with high portions of β‐O‐4‐ethers may be favorable to achieve efficient pretreatment of biomass resulting in high fermentation yields. Often, high syringyl lignins contain higher portions of β‐ether linkages because during cross‐coupling monolignols couple to a syringyl unit (from oligolignols) only through β‐O‐4 linkages (Ralph et al., 2004). However, in our study lignin isolated from stems of genotype OPM‐3, which had the highest S/G ratio, did not contain the highest β‐O‐4 portion.

### 3.3 Phenolic monomers and ferulic acid dehydrodimers and ‐trimers

Grasses usually incorporate ferulic and *p*‐coumaric acid into their cell walls. Ferulic acid is mainly attached to the arabinofuranose side‐chains of arabinoxylans. Formation of ferulate dimers, trimers, or tetramers results in cross‐linked cell wall polysaccharides (Bunzel, 2010). In addition, ferulates play an important role in the formation of cross‐links between polysaccharides and lignin (Jacquet, Pollet, & Lapierre, 1995; Ralph, Grabber, & Hatfield, 1995). Although *p*‐coumaric acid is found to be linked to polysaccharides to a lesser extent, too (Allerdings et al., 2006), it is predominantly attached to lignins (Ralph, 2010). *p*‐Coumaric acid is described to be ester‐linked to the γ‐position of lignins, which indicates the formation of *p*‐coumaric acid‐monolignol conjugates prior to the lignification process (Crestini & Argyropoulos, 1997; Ralph, Hatfield, et al., 1994).

Here, *p*‐coumaric and ferulic acid were identified as the most abundant hydroxycinnamate monomers (Table 6), which is in accordance with literature data (Belmokhtar, Habrant, Lopes Ferreira, & Chabbert, 2013; da Costa et al., 2017; Le Ngoc Huyen et al., 2010; Lygin et al., 2011). The total hydroxycinnamate monomer contents of *Miscanthus* stems of OPM‐3, ‐6, and ‐9 (about 2,102–2,450 mg/100 g dry weight) were about 1.5–2.0‐fold higher than in the leaves of the corresponding genotypes (about 1,196–1,486 mg/100 g dry weight), which results mainly from higher *p*‐coumaric acid contents in the stems of these genotypes. In contrast, contents of total hydroxycinnamate monomers were comparable (about 1720 and 1814 mg/100 g dry weight) in stems and leaves of OPM‐11. *Trans*‐*p*‐coumaric acid was the most abundant phenolic acid in all samples (about 721–1,096 mg/100 g dry weight in the leave fraction; about 1,174–1,914 mg/100 g dry weight in the stem fraction), whereas *trans*‐ferulic acid contents were about 2–3‐fold lower (about 278–530 mg/100 g dry weight). *p*‐Coumaric and ferulic acid were found in their *cis*‐configuration, too. As expected, the portion of *cis*‐configured hydroxycinnamates was higher in the leave fraction, most likely due to light exposure that supports *trans/cis*‐isomerization. However, formation of small amounts of *cis*‐isomers during sample preparation cannot be excluded.

**Table 6:** Contents of cell wall bound, alkali‐extractable hydroxycinnamic acid monomers and diferulic acids (DFA) of different *Miscanthus* genotypes separated into leaves and stems (*n* = 3)

Phenolic monomer (mg/100 g dry weight)	Leaves				Stems			
	OPM‐3	OPM‐6	OPM‐9	OPM‐11	OPM‐3	OPM‐6	OPM‐9	OPM‐11
*trans*‐*p*‐coumaric acid	720.9 (± 59.6)^a^	937.5 (± 19.2)^b^	761.9 (± 28.7)^a^	1,095.5 (± 79.4)^c^	1914.1 ± 57.3^a^	1652.3 (± 48.5)^b^	1810.2 (± 70.9)^a^	1,174.1 (± 9.8)^c^
*cis*‐*p*‐coumaric acid	121.5 (± 0.5)^a^	140.8 (± 13.0)^a,b^	150.6 (± 5.2)^b^	144.5 (± 12.3)^a,b^	51.8 (± 2.6)^a^	38.4 (± 1.4)^b^	44.7 (± 2.2)^c^	37.7 (± 0.5)^b,c^
*trans*‐ferulic acid	320.7 (± 25.6)^a,b^	327.5 (± 7.2)^a^	278.4 (± 9.1)^b^	530.5 (± 40.4)^c^	463.7 (± 5.8)^a^	394.6 (± 10.4)^b^	301.1 (± 12.8)^c^	488.0 (± 3.9)^d^
*cis*‐ferulic acid	32.9 (± 0.2)^a^	34.8 (± 3.0)^a^	30.1 (± 0.9)^a^	43.7 (± 3.0)^b^	20.6 (± 1.1)^a^	16.5 (± 0.8)^b^	15.1 (± 1.6)^b,c^	20.0 (± 0.5)^a^
∑phenolic monomers	1,196.1 (± 85.1)^a^	1,485.6 (± 15.9)^b^	1,221.0 (± 42.7)^a^	1814.2 (± 134.7)^c^	2,450.1 (± 66.5)^a^	2,101.7 (± 59.9)^b^	2,171.2 (± 86.8)^b^	1719.8 (± 13.0)^c^
DFA (mg/100 g dry weight)								
∑8–5‐DFA^a^^,^ b	35.6 (± 1.3)^a^	51.1 (± 5.1)^b^	27.5 (± 2.9)^a^	70.1 (± 2.3)^c^	21.7 (± 1.2)^a^	21.3 (± 0.6)^a^	16.1 (± 0.5)^b^	24.9 (± 2.9)^a^
8‐O‐4‐DFA	17.2 (± 0.5)^a^	19.8 (± 1.2)^b^	17.5 (± 0.3)^a^	30.2 (± 0.4)^c^	4.1 (± 0.2)^a^	5.3 (± 0.2)^b^	3.3 (± 0.1)^c^	5.9 (± 0.3)^d^
5–5‐DFA^c^	9.7 (± 0.5)^a^	10.6 (± 0.8)^a^	10.6 (± 0.5)^a^	15.1 (± 0.1)^b^	2.5 (± 0.1)^a^	2.8 (± 0.1)^a^	1.7 (± 0.1)^b^	3.2 (± 0.2)^c^
8–8‐non‐cyclic‐DFA^c^	–	–	–	–	1.0 (± 0.04)^a^	0.9 (± 0.05)^a^	0.6 (± 0.1)^b^	1.1 (± 0.03)^a^
∑DFA	62.5 (± 1.7)^a^	81.6 (± 7.1)^b^	55.6 (± 3.3)^a^	115.4 (± 2.6)^c^	29.3 (± 1.5)^a^	30.4 (± 0.7)^a,b^	21.7 (± 0.5)^c^	35.1 (± 3.4)^b^

Note

Means labeled with different letters are statistically different (ANOVA, Tukey test, α = 0.05, leaves and stems are tested separately). *p*, para.

a Sum of 8‐5‐cyclic‐, 8‐5‐non‐cyclic‐, and 8‐5‐decarboxylated‐DFA.

b Semiquantitative determination of 8‐5‐coupled DFA contents in *Miscanthus* stems, and of 8‐5‐non‐cyclic‐DFA contents in *Miscanthus* leaves: values were below the tested concentration range, but >limit of quantitation.

c Semiquantitative determination of contents in *Miscanthus* stems: values were below the tested concentration range, but >limit of quantitation.

Significant differences of *p*‐coumaric and ferulic acid contents among the genotypes were observed (ANOVA, Tukey test, *α* = 0.05). Genotype OPM‐11 had the lowest content of *p*‐coumaric acid but the highest ferulic acid content in the quantitatively significant stem fraction. Highest amounts of *p*‐coumaric acid were found in stems of genotypes OPM‐3 and OPM‐9. The opposite trend was observed for the leave fraction. Comparison of the hydroxycinnamate levels of leaves from the different genotypes shows the highest *p*‐coumaric and ferulic acid contents for OPM‐11, but the lowest *p*‐coumaric acid contents for OPM‐3 and OPM‐9.

DFA analysis reveals lower amounts of ferulate dimers compared to the hydroxycinnamate monomer contents (about 63–115 mg DFA/100 g dry weight in *Miscanthus* leaves, and about 22–35 mg DFA/100 g in *Miscanthus* stems; Table 6). Total DFA contents of *Miscanthus* leaves were 2–3‐fold higher than those of *Miscanthus* stems. The most abundant DFA in both fractions was 8–5‐coupled‐DFA (50%–70% of the total DFA content), followed by 8‐O‐4‐DFA (13%–48% of the total DFA content), and 5–5‐DFA (8%–24% of the total DFA content). The content of 8–5‐DFA is given as sum of 8–5‐cyclic‐, 8–5‐non‐cyclic‐, and 8‐5‐decarboxylated‐DFA because 8‐5‐cyclic‐DFA appears to be the only native 8‐5‐coupled dimer in the cell wall. The other two 8‐5‐coupled dimers are suggested to arise during saponification (Bunzel, 2010; Ralph, Quideau, Grabber, & Hatfield, 1994). Small amounts of 8‐8‐non‐cyclic‐DFA (3% of the total DFA) were quantified in *Miscanthu*s stems, too. Although this dimer was also identified in *Miscanthus* leaves, quantitation was not possible due to partial coelution with matrix compounds. Literature data on DFA in *Miscanthus* grass are rarely available (Lygin et al., 2011). Lygin et al. (2011) analyzed various genotypes of *Miscanthus* for their DFA profiles. They found 8‐O‐4‐ and 5‐5‐DFA as most abundant dimers, followed by 8‐5‐ and 8‐8‐DFA. However, they did not distinguish between different forms of 8‐5‐coupled dimers.

Significant differences of the total DFA content and individual DFAs were observed in both leaves and stems (ANOVA, Tukey test, *α* = 0.05, Table 5). OPM‐11 had the highest total DFA contents in both stems (35.1 ± 3.4 mg/100 g dry weight) and leaves (115.4 ± 2.6 mg/100 g dry weight). In contrast, the lowest total DFA contents in both stems (21.7 ± 0.5 mg/100 g dry weight) and leaves (55.6 ± 3.3 mg/100 g dry weight) were analyzed for OPM‐9.

Biomass recalcitrance may also be influenced by the presence of hydroxycinnamates, especially by the formation of cell wall cross‐links. Considering leave/stem ratios, genotype OPM‐11 contained the highest content of total DFAs, whereas the lowest content was found for OPM‐9 (approximately half of the DFA content of OPM‐11).

## 4 CONCLUSION

The recalcitrance of lignocellulosic biomass against hydrolysis and fermentation is influenced by its gross composition, structural features of its cell wall polymers, and their interactions. Here, different parts of the *Miscanthus* plant (leaves vs. stems) and different genotypes (*Miscanthus sacchariflorus*,* Miscanthus sinensis* × *Miscanthus sacchariflorus* hybrid, *Miscanthus* × *giganteus*,* Miscanthus sinensis* “Goliath”) were shown to partially differ in the overall cell wall composition and in structural details of the cell wall polymers demonstrating some genetic variability of *Miscanthus*. Although specific parameters such as reduced lignin contents, specific types of linkages within the lignin polymers, etc., are generally associated with biomass saccharification efficiency, an unambiguous prediction of the fermentation yields of biomass from the different *Miscanthus* genotypes appears difficult as none of the studied genotypes combines all favorable traits. For example, the high lignin content (determined as ABSL) of *Miscanthus* × *giganteus* is supposed to reduce the saccharification efficiency of this genotype. However, lignins isolated from this genotype contained the highest portions of β‐O‐4‐linkages, which may be more easily cleaved during pretreatment processes to efficiently reduce the lignin content. Because Klason lignin and ABSL contents of *Miscanthus* stems were higher than lignin contents of *Miscanthus* leaves, the recalcitrance of stems is potentially higher than that of leaves. However, *Miscanthus* leaves contained higher DFA contents which is suggested to increase biomass recalcitrance.

Although the usefulness of cell wall composition data for the evaluation of biomass utilization is undisputed, this data need to considered in conjunction with potential biomass pretreatments to combine plant material and processes for biochemical use in an optimal way.
